# Noncircular Distributed Source DOA Estimation with Nested Arrays via Reduced-Dimension MUSIC

**DOI:** 10.3390/s24206653

**Published:** 2024-10-15

**Authors:** Kaiyuan Chen, Weiyang Chen, Jiaqi Li

**Affiliations:** College of Electronic and Information Engineering, Nanjing University of Aeronautics and Astronautics, Nanjing 211106, China; weiweigenes@nuaa.edu.cn (W.C.); lijiaqi2000@outlook.com (J.L.)

**Keywords:** DOA estimation, coherently distributed source, nested array, reduced-dimension MUSIC

## Abstract

This paper focuses on the direction-of-arrival (DOA) estimation for noncircular coherently distributed (CD) sources with nested arrays. Usually, for point sources, sparse arrays have the potential to improve the estimation performance of algorithms by obtaining more degrees of freedom. However, algorithms have to be reconsidered for CD sources with sparse arrays and many problems arise. One thorny problem is the disappearance of displacement invariance of the virtual array manifold constructed by the virtualization technique. To deal with this issue, a nested array processing method for CD sources transmitting noncircular signals is proposed in this paper. Firstly, we construct the virtual sum-and-difference co-array by leveraging the noncircular quality of signals with a nested array. Then, an approximation is made to degrade CD sources into point sources. In this way, spatial smoothing techniques can be applied to restore the rank. Finally, in order to reduce the complexity, we modify the reduced-dimension MUSIC to estimate DOAs through a one-dimensional peak-searching procedure. The simulation results prove the superiority of our algorithm against other competitors.

## 1. Introduction

Direction-of-arrival (DOA) estimation is one of the most important research contents in array signal processing, which has many applications in radar and sonar systems [1,2]. At the very beginning, researchers mainly focused on methods such as conventional beamforming and maximum likelihood. Ever since the multiple signal classification (MUSIC) algorithm [3] was proposed, studies on DOA estimation have entered a new era. Many high-resolution algorithms have been developed based on the idea of signal and noise subspace, e.g., estimation of signal parameters via rotational invariance techniques (ESPRIT) [4]. Even though the accuracy of these high-performance algorithms is attractive, their computational burden becomes a trouble, especially for some practical systems. To solve this problem in practice, some simple techniques, such as phase interferometry (PI) [5], together with the design of particular systems, are proposed [6].

### 1.1. Prior Art

In many works, it is often assumed that the signal sources are point sources for the sake of simplicity [7,8]. However, the multipath propagation in the real world caused by reflection and scattering of the surrounding objects near the source could lead to serious performance deterioration of algorithms based on this ideal model [9]. As a result, research has been conducted to solve this issue. Some representative distributed source models are proposed by S. Valaee [10]. According to his work, distributed sources can be sorted according to the degrees of correlation among signal components. As one of the most commonly used abstractions, the coherently distributed (CD) source refers to the one whose signal components are fully correlated. Methods such as MUSIC and ESPRIT are reconsidered and adapted to solve DOA estimation problems with distributed sources [10,11,12,13].

A direction-finding algorithm usually comes with a specific array configuration. Basically, a uniform linear array (ULA) is leveraged for the estimation program. However, considering the relatively small spacing between sensors and limited degrees of freedom (DOFs), various sparse array structures have been studied in recent years. Coprime array [14,15] and nested array (NA) [16] are two classical and practical configurations. Ways to process signal information received by these arrays come together [17,18,19]. A popular one is to construct a virtual array using second-order statistics (SOSs) of the signal [18]. By carefully crafting the SOSs, a single-snapshot signal of a much longer virtual array can be constructed. Rank-restoring methods like spatial smoothing technique (SS) [18,20] could be applied to form a nonsingular signal matrix. In this way, the array aperture is enlarged, and a higher number of DOFs is obtained.

Complex-valued signals play an important role in array signal processing [21]. Basically, signals can be classified into two types based on the circularity property, i.e., circular signals and noncircular signals. A parameter called noncircularity rate is used to quantitatively characterize the circularity of the signal [22]. Traditionally, circular signals are considered in DOA estimation simulation. Some communication signals like 8 phase-shift keying modulated signals are modeled as circular signals. Noncircular signals, such as binary phase-shift keying modulated signals, are also very common in communication systems, which are expected to increase DOFs further. Therefore, when considering a noncircular source, algorithms developed for circular signals need to be modified to exploit the additional information brought by noncircular phases [22,23]. This idea is applicable for DOA estimation with sparse arrays [24,25]. Traditionally, only the covariance matrix is employed to construct the virtual array, which is called the difference co-array (DCA). With noncircular signals, more DOFs should be gained. Hence, researchers turn their attention to the elliptic covariance matrix of the signal. Algorithm (SD-RD-MUSIC) with a special virtual array called sum-and-difference co-array (SDCA) is designed by Y. Wang in [24], where the reduced-dimension MUSIC (RD-MUSIC) [26] is applied to decouple the estimation of parameters. Sum co-arrays (SCAs) created by the elliptic covariance matrix help to provide extra DOFs.

### 1.2. Motivations and Contributions

Although it seems that the research on algorithms aimed at DOA estimation with sparse arrays has been well conducted, relevant studies for distributed sources are still insufficient. Normal rank-restoring methods fail in this case because the virtual array manifold does not have displacement invariance quality anymore. To circumvent this problem, a novel spatial smoothing (NSS) technique is studied in [27] with a distributed source model introduced in [28]. Sources are degraded into conventional point source models with prior knowledge about the spread extent of sources. In this paper, we would like to develop an algorithm to conquer the DOA estimation problem with sparse arrays with the type of CD source model provided in [10] due to its popularity. Also, we hope to come up with a way to leverage noncircular quality in this scenario at the same time. Thus, the DOA estimation for a CD source proposed in [10] emitting noncircular signals with a nested array is considered, and an algorithm (MSD-RD-MUSIC) modified from SD-RD-MUSIC to deal with this issue is proposed.

The main contributions of this paper are as follows:

1. We analyze the similarity among the sources in a certain parameter and degrade the CD sources into point sources based on this nature. Then, a rank-restoring method like a spatial smoothing technique could be applied to form a full-rank signal matrix.
2. The feature of noncircular signals is taken advantage of by a nested array to form a DCA and two SCAs, which are well used to construct a longer virtual ULA. More DOFs are obtained compared with other algorithms [11,13,24,27]. The outstanding performance of the proposed algorithm is verified in simulations.
3. Based on the idea of RD-MUSIC, we develop the MSD-RD-MUSIC to estimate the DOA parameter through a one-dimensional peak-searching procedure, which significantly lowers the computational complexity.

### 1.3. Organization and Notations

The rest of this paper is organized as follows. In Section 2, we illustrate the mathematical model, including the CD source model we adopt, nested array structure, noncircular signals, and the SDCA model. In Section 3, our proposed MSD-RD-MUSIC is presented together with a virtual array construction method. DOFs and the computational complexity of several different algorithms are analyzed in Section 4 to illustrate the advantages of MSD-RD-MUSIC. Numerical simulations are carried out to show the superiority concretely in Section 5. Finally, in Section 6, the conclusion is drawn.

In the following, vectors and matrices are denoted by lower-case and upper-case boldface symbols, respectively. Superscripts ${\left(\cdot \right)}^{T}$, ${\left(\cdot \right)}^{H}$, and ${\left(\cdot \right)}^{*}$ stand for transpose, conjugate transpose, and conjugation respectively. We define that $\langle a,b\rangle ≜\left\{\left.x\right|a⩽x⩽b,x\in Z\right\}$. The determinant of a matrix is represented by $\det\left(\cdot \right)$. Operator ⊙ and ⊗ stand for Khatri–Rao and Kronecker product. A diagonal matrix with entries from vector x is represented by $\mathrm{diag}\left(x\right)$. The *m*th element of vector x is denoted by ${\left[x\right]}_{m}$. $E\left[\cdot \right]$ outputs the expectation. The column vectorization of matrix A is returned by $\mathrm{vec}\left(A\right)$. The imaginary unit is denoted by symbol *j*.

## 2. Mathematical Model

### 2.1. Coherently Distributed Source

The narrowband signals emitted from *K* far-field sources are received by a linear array of *N* omnidirectional sensors. We assume that the signals are reflected and scattered by some objects around the sources and that the signal components from one source are continuously distributed in the spatial domain and fully correlated with each other. Then, the model of the received signal is described as [10]

(1) $$\begin{array}{c} \begin{array}{c} \begin{array}{cc} x(t) & =\sum_{k=1}^{K}\int_{-\pi /2}^{\pi /2}a\left(\theta \right)s_{k}\left(\theta ,t;\eta_{k}\right)d\theta +n\left(t\right)\\ \; & =\sum_{k=1}^{K}\int_{-\pi /2}^{\pi /2}a\left(\theta \right)\gamma_{k}\left(t\right)h\left(\theta ,\eta_{k}\right)d\theta +n\left(t\right) \end{array} \end{array} \end{array}$$

where a(θ) is the steering vector, $s_{k}\left(\theta ,t;\eta_{k}\right)=\gamma_{k}\left(t\right)h\left(\theta ,\eta_{k}\right)$ and $\eta_{k}={\left[\theta_{k},\sigma_{k}\right]}^{T}$ stand for the angular signal density and the unknown parameter vector for the *k*th source. $\theta_{k}$ and $\sigma_{k}$ denote the DOA and the angular spread, respectively. $\gamma_{k}\left(t\right)$ reflects the feature of the source in the time domain, and $h(\theta ,\eta_{k})$ is the deterministic angular density function. $n\left(t\right)$ is the zero-mean, complex Gaussian additive noise with power $\sigma_{n}^{2}$.

For any array configuration, the positions of each array element are collected in the position vector d. We separate the unit intersensor spacing $d_{0}$ from d, and we have $d=zd_{0}$. z is the index position vector of the array elements. For the sake of convenience, the distance between the position of the *n*th sensor and the reference sensor is written as $z_{n}={\left[z\right]}_{n}$. Then, we know that

(2) $$\begin{array}{c} \begin{array}{c} a\left(\theta \right)={\left[e^{jz_{1}\Delta \sin\theta },e^{jz_{2}\Delta \sin\theta },\ldots ,e^{jz_{K}\Delta \sin\theta }\right]}^{T} \end{array} \end{array}$$

where $\Delta =2\pi d_{0}/\lambda$, with λ being the wavelength of the signal.

We denote the generalized steering vector for a CD source as

(3) $$\begin{array}{c} \begin{array}{c} b\left(\eta_{k}\right)=\int_{-\pi /2}^{\pi /2}a\left(\theta \right)h\left(\theta ,\eta_{k}\right)d\theta \end{array} \end{array}$$

and the generalized array manifold for CD sources is defined as

(4) $$\begin{array}{c} \begin{array}{c} B=\left[b\left(\eta_{1}\right),b\left(\eta_{2}\right),\ldots ,b\left(\eta_{K}\right)\right] \end{array} \end{array}$$

We consider the most commonly used assumption for the CD sources in this paper and assume that the deterministic function $h(\theta ,\eta_{k})$ follows Gaussian distribution [29], which means

(5) $$\begin{array}{c} \begin{array}{c} h\left(\theta ,\eta_{k}\right)=\frac{1}{{\left(2\pi \right)}^{1/2}\sigma_{k}}e^{-\frac{{\left(\theta -\theta_{k}\right)}^{2}}{2\sigma_{k}^{2}}} \end{array} \end{array}$$

Then, according to Taylor’s formula [29,30], when the angular spread of the source is relatively small, the closed form of the steering vector in (3) is

(6) $$\begin{array}{c} \begin{array}{c} {\left[b\left(\eta_{k}\right)\right]}_{m}={\left[a\left(\theta_{k}\right)\right]}_{m}e^{-\frac{1}{2}{\left(z_{m}\Delta \cos\left(\theta_{k}\right)\sigma_{k}\right)}^{2}} \end{array} \end{array}$$

We define a complex parameter $\rho_{k}$ as a new unknown parameter calculated from $\theta_{k}$ and $\sigma_{k}$:

(7) $$\begin{array}{c} \begin{array}{c} \rho_{k}=\rho \left(\theta_{k},\sigma_{k}\right)=e^{-\frac{1}{2}{\left(\Delta \cos\;\;\left(\theta_{k}\right)\sigma_{k}\right)}^{2}} \end{array} \end{array}$$

Then, we substitute (7) into (6) in order to highlight the relationship between the steering vector $b\left(\eta_{k}\right)$ and the index vector of sensors in the array. Equation (6) is rewritten as (8) in a simplified version:

(8) $$\begin{array}{c} \begin{array}{c} {\left[b\left(\eta_{k}\right)\right]}_{m}={\left[a\left(\theta_{k}\right)\right]}_{m}\rho_{k}^{z_{m}^{2}} \end{array} \end{array}$$

Finally, the model of the received signal is given as

(9) $$\begin{array}{c} \begin{array}{c} x\left(t\right)=\mathrm{Bs}\left(t\right)+n\left(t\right) \end{array} \end{array}$$

where $s\left(t\right)={\left[\gamma_{1}\left(t\right),\gamma_{2}\left(t\right),\cdots ,\gamma_{K}\left(t\right)\right]}^{T}$.

### 2.2. Noncircular Signals

Based on the definition of noncircular signals provided in [31], the strictly noncircular signal is considered here. In this way, signal $s\left(t\right)$ satisfies

(10) $$\begin{array}{c} \begin{array}{c} s\left(t\right)=\Phi s_{R}\left(t\right) \end{array} \end{array}$$

where $\Phi =\mathrm{diag}\left(e^{j\varphi_{1}},e^{j\varphi_{2}},\ldots ,e^{j\varphi_{K}}\right)$, with $\varphi_{k}$ being the noncircular phase of the *k*th source, and $s_{R}\left(t\right)$ is the real part of the signals. Substitute the form of the noncircular signals into (9), and the equivalent array manifold for CD sources emitting noncircular signals is given as

(11) $$\begin{array}{c} \begin{array}{c} C=B\Phi =\left[c\left(\eta_{1}\right),c\left(\eta_{2}\right),\ldots ,c\left(\eta_{K}\right)\right] \end{array} \end{array}$$

To fully exploit the noncircular features of the signals, subsequent signal processing procedures will be based on the extended received signals:

(12) $$\begin{array}{c} \begin{array}{c} x_{e}\left(t\right)=\left[\begin{array}{c} x\left(t\right)\\ x^{*}\left(t\right) \end{array}\right]=\left[\begin{array}{c} C\\ C^{*} \end{array}\right]s_{R}\left(t\right)+\left[\begin{array}{c} n(t)\\ n^{*}\left(t\right) \end{array}\right] \end{array} \end{array}$$

The covariance matrix of the extended received signal is

(13) $$\begin{array}{c} \begin{array}{c} R_{x_{e}}=E\left[x_{e}\left(t\right)x_{e}^{H}\left(t\right)\right]=E\left[\begin{array}{cc} x\left(t\right)x^{H}\left(t\right) & x\left(t\right)x^{T}\left(t\right)\\ x^{*}\left(t\right)x^{H}\left(t\right) & x^{*}\left(t\right)x^{T}\left(t\right) \end{array}\right]=\left[\begin{array}{cc} R_{11} & R_{12}\\ R_{21} & R_{22} \end{array}\right] \end{array} \end{array}$$

However, in practice, the covariance matrix can only be estimated from finite snapshots:

(14) $$\begin{array}{c} \begin{array}{c} {\hat{R}}_{x_{e}}=\frac{1}{L}\sum_{t=1}^{L}x_{e}\left(t\right)x_{e}^{H}\left(t\right) \end{array} \end{array}$$

### 2.3. Nested Array and Sum-and-Difference Co-Array

**Figure 1:**
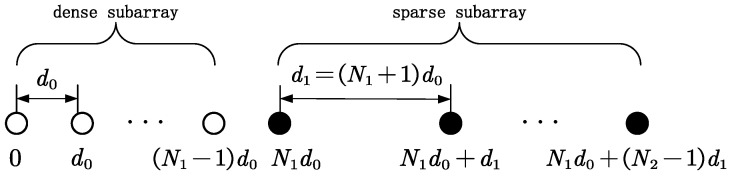
Two-level nested array.

As is shown in Figure 1, a two-level NA [16] is employed in this paper, which contains a dense subarray with $N_{1}$ sensors and a sparse subarray with $N_{2}$ sensors, respectively. So, the number of sensors is $N=N_{1}+N_{2}$, and the spacings of the two subarrays are $d_{0}$ and $d_{1}=\left(N_{1}+1\right)d_{0}$. The index position vector is expressed as

(15) $$\begin{array}{c} \begin{array}{c} z={\left[0,1,\ldots ,N_{1}-1,N_{1},N_{1}+\left(N_{1}+1\right),\ldots ,N_{1}+\left(N_{2}-1\right)\left(N_{1}+1\right)\right]}^{T} \end{array} \end{array}$$

In handling the SOSs of signals received by the NA and constructing virtual sensors, vectorization of the covariance matrix is inevitable. We divide the covariance matrix into four parts in (13) and vectorize each part separately. The results are given as

(16) $$\begin{array}{c} \begin{array}{c} \begin{array}{cc} u_{1} & =\mathrm{vec}\left(R_{11}\right)=\mathrm{vec}\left({\mathrm{CR}}_{ss}C^{H}+\sigma_{n}^{2}I\right)=\left(C^{*}⊙C\right)p+\sigma_{n}^{2}{\tilde{1}}_{n}=\left(B^{*}⊙B\right)p+\sigma_{n}^{2}{\tilde{1}}_{n}\\ \; & =\left[b^{*}\left(\eta_{1}\right)⊗b\left(\eta_{1}\right),b^{*}\left(\eta_{2}\right)⊗b\left(\eta_{2}\right),\ldots ,b^{*}\left(\eta_{K}\right)⊗b\left(\eta_{K}\right)\right]p+\sigma_{n}^{2}{\tilde{1}}_{n} \end{array} \end{array} \end{array}$$

(17) $$\begin{array}{c} \begin{array}{c} \begin{array}{cc} u_{2} & =\mathrm{vec}\left(R_{12}\right)=\mathrm{vec}\left({\mathrm{CR}}_{ss}C^{T}\right)=\left(C⊙C\right)p\\ \; & =\left[b\left(\eta_{1}\right)⊗b\left(\eta_{1}\right)e^{j2\varphi_{1}},b\left(\eta_{2}\right)⊗b\left(\eta_{2}\right)e^{j2\varphi_{2}},\ldots ,b\left(\eta_{K}\right)⊗b\left(\eta_{K}\right)e^{j2\varphi_{K}}\right]p \end{array} \end{array} \end{array}$$

(18) $$\begin{array}{c} \begin{array}{c} \begin{array}{cc} u_{3} & =\mathrm{vec}\left(R_{21}\right)=\mathrm{vec}\left(C^{*}R_{ss}C^{H}\right)=\left(C^{*}⊙C^{*}\right)p\\ \; & =\left[b^{*}\left(\eta_{1}\right)⊗b^{*}\left(\eta_{1}\right)e^{-j2\varphi_{1}},b^{*}\left(\eta_{2}\right)⊗b^{*}\left(\eta_{2}\right)e^{-j2\varphi_{2}},\ldots ,b^{*}\left(\eta_{K}\right)⊗b^{*}\left(\eta_{K}\right)e^{-j2\varphi_{K}}\right]p \end{array} \end{array} \end{array}$$

(19) $$\begin{array}{c} \begin{array}{c} \begin{array}{cc} u_{4} & =\mathrm{vec}\left(R_{22}\right)=\mathrm{vec}\left(C^{*}R_{ss}C^{T}+\sigma_{n}^{2}I\right)=\left(C⊙C^{*}\right)p+\sigma_{n}^{2}{\tilde{1}}_{n}=\left(B⊙B^{*}\right)p+\sigma_{n}^{2}{\tilde{1}}_{n}\\ \; & =\left[b\left(\eta_{1}\right)⊗b^{*}\left(\eta_{1}\right),b\left(\eta_{2}\right)⊗b^{*}\left(\eta_{2}\right),\ldots ,b\left(\eta_{K}\right)⊗b^{*}\left(\eta_{K}\right)\right]p+\sigma_{n}^{2}{\tilde{1}}_{n} \end{array} \end{array} \end{array}$$

where $p={\left[\sigma_{s1}^{2},\sigma_{s2}^{2},\ldots ,\sigma_{sK}^{2}\right]}^{T}$, with $\sigma_{sk}^{2}$ being the power of the *k*th source, ${\tilde{1}}_{n}={\left[e_{1}^{T},e_{2}^{T},\ldots ,e_{N}^{T}\right]}^{T}$, $e_{n}$ being a column vector with all zeros but a 1 at the *n*th position, and $R_{ss}=E\left[s_{R}\left(t\right)s_{R}^{H}\left(t\right)\right]=E\left[s_{R}\left(t\right)s_{R}^{T}\left(t\right)\right]$ being the covariance matrix of the signals arriving at the NA.

According to [14,24], if we only focus on the phases, each row of $u_{1}$, $u_{2}$, $u_{3}$, $u_{4}$ would correspond to the signal received by virtual sensors at a certain position. Thus, by extracting continuous parts of the rows from $u_{1}$ and $u_{4}$, we can obtain the virtual signal received by a DCA. We can have virtual signals received by SCAs by extracting rows from $u_{2}$ and $u_{3}$. The index position set of the DCA is defined as

(20) $$\begin{array}{c} \begin{array}{c} D=\left\{z_{m}-z_{n}|m,n=1,2,\ldots ,N\right\} \end{array} \end{array}$$

The set of the positive and negative SCA are represented as

(21) $$\begin{array}{c} \begin{array}{c} S_{+}=\left\{z_{m}+z_{n}|m,n=1,2,\ldots ,N\right\} \end{array} \end{array}$$

and

(22) $$\begin{array}{c} \begin{array}{c} S_{-}=\left\{-z_{m}-z_{n}|m,n=1,2,\ldots ,N\right\} \end{array} \end{array}$$

Remove the repeated items, continuous parts of the DCA and SCAs are

(23) $$\begin{array}{c} \begin{array}{c} \tilde{D}=\langle -R_{1},R_{1}\rangle \end{array} \end{array}$$

and

(24) $$\begin{array}{c} \begin{array}{c} \tilde{S}=\langle -R_{3},-R_{2}\rangle \cup \langle R_{2},R_{3}\rangle \end{array} \end{array}$$

where $R_{1}=N_{1}N_{2}+N_{2}-1$, $R_{2}=0$, $R_{3}=N_{1}N_{2}+N_{1}+N_{2}-1$ for the two-level NA employed [24].

## 3. Proposed Algorithm

In this section, we propose the DOA estimation method (MSD-RD-MUSIC) for CD sources emitting noncircular signals with a two-level NA.

### 3.1. Virtual Array Construction

Given the fact that DCAs constructed from $u_{1}$ and $u_{4}$ are the same, we only extract the continuous part from $u_{1}$, $u_{2}$, and $u_{3}$. The virtual received signals of DCA, positive SCA, and negative SCA are denoted by ${\tilde{u}}_{1}$, ${\tilde{u}}_{2}$, and ${\tilde{u}}_{3}$, respectively. We have

(25) $$\begin{array}{c} \begin{array}{c} {\tilde{u}}_{1}={\tilde{C}}_{1}p+\sigma_{n}^{2}{\tilde{e}}_{n} \end{array} \end{array}$$

(26) $$\begin{array}{c} \begin{array}{c} {\tilde{u}}_{2}={\tilde{C}}_{2}p \end{array} \end{array}$$

(27) $$\begin{array}{c} \begin{array}{c} {\tilde{u}}_{3}={\tilde{C}}_{3}p \end{array} \end{array}$$

where ${\tilde{e}}_{n}$ is a column vector with a 1 in the center of it and zeros in the rest positions. Notice that ${\tilde{u}}_{2}$ and ${\tilde{u}}_{3}$ are extracted using the opposite order. Thus, the *k*th steering vector of the DCA and SCAs are expressed as

(28) $$\begin{array}{c} \begin{array}{c} {\tilde{c}}_{1}\left(\eta_{k}\right)={\left[\rho_{k}^{{\tilde{\dot{z}}}_{1}}e^{j\left(-R_{1}\right)\Delta \sin\theta_{k}},\rho_{k}^{{\tilde{\dot{z}}}_{2}}e^{j\left(-R_{1}+1\right)\Delta \sin\theta_{k}},\ldots ,\rho_{k}^{{\tilde{\dot{z}}}_{2R_{1}+1}}e^{jR_{1}\Delta \sin\theta_{k}}\right]}^{T} \end{array} \end{array}$$

(29) $$\begin{array}{c} \begin{array}{c} {\tilde{c}}_{2}\left(\eta_{k},\varphi_{k}\right)={\left[\rho_{k}^{{\tilde{\ddot{z}}}_{1}},\rho_{k}^{{\tilde{\ddot{z}}}_{2}}e^{j\Delta \sin\theta_{k}},\ldots ,\rho_{k}^{{\tilde{\ddot{z}}}_{R_{3}+1}}e^{jR_{3}\Delta \sin\theta_{k}}\right]}^{T}e^{j2\varphi_{k}} \end{array} \end{array}$$

(30) $$\begin{array}{c} \begin{array}{c} {\tilde{c}}_{3}\left(\eta_{k},\varphi_{k}\right)={\left[\rho_{k}^{{\tilde{\ddot{z}}}_{R_{3}+1}}e^{j\left(-R_{3}\right)\Delta \sin\theta_{k}},\rho_{k}^{{\tilde{\ddot{z}}}_{R_{3}}}e^{j\left(-R_{3}+1\right)\Delta \sin\theta_{k}},\ldots ,\rho_{k}^{{\tilde{\ddot{z}}}_{1}}\right]}^{T}e^{-j2\varphi_{k}} \end{array} \end{array}$$

where ${\tilde{\ddot{z}}}_{m}$ ($m=1,2,\ldots ,R_{3}+1$) and ${\tilde{\dot{z}}}_{n}$ ($n=1,2,\ldots ,2R_{1}+1$) are the powers of parameter $\rho_{k}$ corresponding to the continuous phases of the virtual DCA and SCAs.

We assume that the synthetic parameter $\rho_{k}$ are the same for all sources involved, i.e., $\rho_{1}=\rho_{2}=\ldots =\rho_{K}=\rho_{0}$. This assumption is similar to [27], however, with a different source model [10,28].

**Remark 1.**  *It is fairly rational to come up with such an assumption and perform derivation with it, because, for the Gaussian CD sources considered in this paper, the spread of sources is relatively small [29], which means parameter $\rho_{k}$ would be quite similar. This point can be verified through calculation. For example, for spread not greater than 5° and intersensor spacing $d_{0}=\lambda /4$, $\rho_{k}$ lies between 0.99 and 1. Therefore, through approximation, $\rho_{k}$ (for k = 1,2, …,K) can be viewed as the same and known. Simulation results prove that the error caused by this approximation is small.*

Diagonal matrices reflecting the influence of $\rho_{0}$ on the constructed virtual single-snapshot output are given as

(31) $$\begin{array}{c} \begin{array}{c} \Psi_{1}=\mathrm{diag}\left(\rho_{0}^{{\tilde{\dot{z}}}_{1}},\rho_{0}^{{\tilde{\dot{z}}}_{2}},\ldots ,\rho_{0}^{{\tilde{\dot{z}}}_{2R_{1}+1}}\right) \end{array} \end{array}$$

(32) $$\begin{array}{c} \begin{array}{c} \Psi_{2}=\mathrm{diag}\left(\rho_{0}^{{\tilde{\ddot{z}}}_{1}},\rho_{0}^{{\tilde{\ddot{z}}}_{2}},\ldots ,\rho_{0}^{{\tilde{\ddot{z}}}_{R_{3}+1}}\right) \end{array} \end{array}$$

(33) $$\begin{array}{c} \begin{array}{c} \Psi_{3}=\mathrm{diag}\left(\rho_{0}^{{\tilde{\ddot{z}}}_{R_{3}+1}},\rho_{0}^{{\tilde{\ddot{z}}}_{R_{3}}},\ldots ,\rho_{0}^{{\tilde{\ddot{z}}}_{1}}\right) \end{array} \end{array}$$

Influence of the parameter $\rho_{0}$ can be removed by premultiplying ${\tilde{u}}_{1}$, ${\tilde{u}}_{2}$, and ${\tilde{u}}_{3}$ by the inverse of $\Psi_{1}$, $\Psi_{2}$, and $\Psi_{3}$, and the transformation is expressed as

(34) $$\begin{array}{c} \begin{array}{c} \begin{array}{cc} u_{1}^{\prime } & =\Psi_{1}^{-1}{\tilde{u}}_{1}=\Psi_{1}^{-1}{\tilde{C}}_{1}p+\sigma_{n}^{2}\Psi_{1}^{-1}{\tilde{e}}_{n}\\ \; & =C_{1}^{\prime }p+\sigma_{n}^{2}\Psi_{1}^{-1}{\tilde{e}}_{n} \end{array} \end{array} \end{array}$$

(35) $$\begin{array}{c} \begin{array}{c} u_{2}^{\prime }=\Psi_{2}^{-1}{\tilde{u}}_{2}=\Psi_{2}^{-1}{\tilde{C}}_{2}p=C_{2}^{\prime }p \end{array} \end{array}$$

(36) $$\begin{array}{c} \begin{array}{c} u_{3}^{\prime }=\Psi_{3}^{-1}{\tilde{u}}_{3}=\Psi_{3}^{-1}{\tilde{C}}_{3}p=C_{3}^{\prime }p \end{array} \end{array}$$

and the steering vectors for the *k*th source become

(37) $$\begin{array}{c} \begin{array}{c} c_{1}^{\prime }\left(\theta_{k}\right)={\left[e^{j\left(-R_{1}\right)\Delta \sin\theta_{k}},e^{j\left(-R_{1}+1\right)\Delta \sin\theta_{k}},\ldots ,e^{jR_{1}\Delta \sin\theta_{k}}\right]}^{T} \end{array} \end{array}$$

(38) $$\begin{array}{c} \begin{array}{c} c_{2}^{\prime }\left(\theta_{k},\varphi_{k}\right)={\left[1,e^{j\Delta \sin\theta_{k}},\ldots ,e^{jR_{3}\Delta \sin\theta_{k}}\right]}^{T}e^{j2\varphi_{k}} \end{array} \end{array}$$

(39) $$\begin{array}{c} \begin{array}{c} c_{3}^{\prime }\left(\theta_{k},\varphi_{k}\right)={\left[e^{j\left(-R_{3}\right)\Delta \sin\theta_{k}},e^{j\left(-R_{3}+1\right)\Delta \sin\theta_{k}},\ldots ,1\right]}^{T}e^{-j2\varphi_{k}} \end{array} \end{array}$$

If we want to eliminate the negative side effect caused by $\Psi_{1}^{-1}$ on the noise ${\tilde{e}}_{n}$, i.e., make $\Psi_{1}^{-1}{\tilde{e}}_{n}={\tilde{e}}_{n}$, ${\tilde{\dot{z}}}_{R_{1}+1}$ is expected to be zero, because there is only one nonzero entry in the center of ${\tilde{e}}_{n}$. It can be satisfied easily because the first element of $b^{*}\left(\eta_{k}\right)⊗b\left(\eta_{k}\right)$ in (16) is $\rho_{k}^{0}$, which just meets the requirement. So, we draw the first entry of $u_{1}$ and put it on the $\left(R_{1}+1\right)$th position in ${\tilde{u}}_{1}$. Nothing needs to be performed for the extraction of $u_{2}$ and $u_{3}$, because ${\tilde{u}}_{2}$ and ${\tilde{u}}_{3}$ do not involve the noise part, which means the multiplication of $\Psi_{2}^{-1}$ and $\Psi_{3}^{-1}$ is not supposed to have much influence.

**Figure 2:**
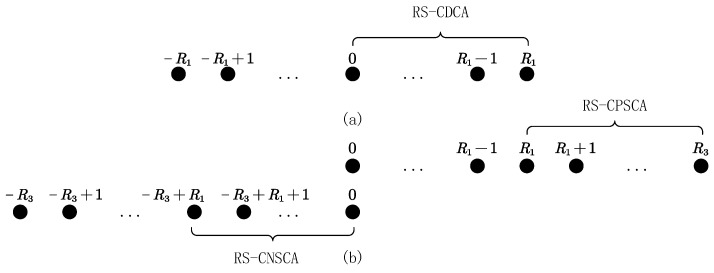
(**a**) Continuous part of the difference co-array. (**b**) Continuous part of the sum co-arrays.

As illustrated in Figure 2, we separate each of the continuous co-arrays into $\left(R_{1}+1\right)$ overlapping subarrays. The reference subarrays of the continuous part of the DCA, positive SCA, and negative SCA are denoted by RS-CDCA, RS-CPSCA, and RS-CNSCA in Figure 2.

The sensors index position of the *i*th subarray of the DCA ranges from (1−i) to $\left(1-i+R_{1}\right)$, and the output of this subarray is given as

(40) $$\begin{array}{c} \begin{array}{c} u_{1i}^{\prime }=C_{11}^{\prime }H^{i-1}p+\sigma_{n}^{2}e_{i}\;,\;i=1,2,\ldots ,R_{1}+1 \end{array} \end{array}$$

where $H=\mathrm{diag}\left(e^{-j\Delta \sin\theta_{1}},e^{-j\Delta \sin\theta_{2}},\ldots ,e^{-j\Delta \sin\theta_{K}}\right)$, and $C_{11}^{\prime }$ is the array manifold of the reference subarray of the continuous part of the DCA. By stacking $u_{11}^{\prime }$, $u_{12}^{\prime }$, …, $u_{1(R_{1}+1)}^{\prime }$ together [24], we get

(41) $$\begin{array}{c} \begin{array}{c} U_{1}^{\prime }=\left[u_{11}^{\prime },u_{12}^{\prime },\ldots ,u_{1\left(R_{1}+1\right)}^{\prime }\right]=C_{11}^{\prime }\left[p,\mathrm{Hp},\ldots ,H^{R_{1}}p\right]+\sigma_{n}^{2}I \end{array} \end{array}$$

which can be regarded as the received signal of RS-CDCA and its steering vector is

(42) $$\begin{array}{c} \begin{array}{c} c_{11}^{\prime }\left(\theta_{k}\right)={\left[1,e^{j\Delta \sin\theta_{k}},\ldots ,e^{jR_{1}\Delta \sin\theta_{k}}\right]}^{T} \end{array} \end{array}$$

The same steps are followed for $u_{2}^{\prime }$ and $u_{3}^{\prime }$. The positions of the sensors in the *i*th subarray of positive and negative SCA are from $\left(R_{1}+1-i\right)$ to $\left(R_{3}+1-i\right)$ and from $\left(-R_{3}+R_{1}+1-i\right)$ to (1−i). The output of the *i*th subarrays for continuous SCAs are expressed as

(43) $$\begin{array}{c} \begin{array}{c} u_{2i}^{\prime }=C_{21}^{\prime }H^{i-1}p \end{array} \end{array}$$

(44) $$\begin{array}{c} \begin{array}{c} u_{3i}^{\prime }=C_{31}^{\prime }H^{i-1}p \end{array} \end{array}$$

with $C_{21}^{\prime }$ and $C_{31}^{\prime }$ being the array manifold of the reference subarrays. Then, we stack the output of these subarrays together:

(45) $$\begin{array}{c} \begin{array}{c} U_{2}^{\prime }=\left[u_{21}^{\prime },u_{22}^{\prime },\ldots ,u_{2\left(R_{1}+1\right)}^{\prime }\right]=C_{21}^{\prime }\left[p,\mathrm{Hp},\ldots ,H^{R_{1}}p\right] \end{array} \end{array}$$

(46) $$\begin{array}{c} \begin{array}{c} U_{3}^{\prime }=\left[u_{31}^{\prime },u_{32}^{\prime },\ldots ,u_{3\left(R_{1}+1\right)}^{\prime }\right]=C_{31}^{\prime }\left[p,\mathrm{Hp},\ldots ,H^{R_{1}}p\right] \end{array} \end{array}$$

Steering vectors for RS-CPSCA and RS-CNSCA are given as

(47) $$\begin{array}{c} \begin{array}{c} c_{21}^{\prime }\left(\theta_{k},\varphi_{k}\right)={\left[e^{jR_{1}\Delta \sin\theta_{k}},e^{j\left(R_{1}+1\right)\Delta \sin\theta_{k}},\ldots ,e^{jR_{3}\Delta \sin\theta_{k}}\right]}^{T}e^{j2\varphi_{k}} \end{array} \end{array}$$

(48) $$\begin{array}{c} \begin{array}{c} c_{31}^{\prime }\left(\theta_{k},\varphi_{k}\right)={\left[e^{j\left(-R_{3}+R_{1}\right)\Delta \sin\theta_{k}},e^{j\left(-R_{3}+R_{1}+1\right)\Delta \sin\theta_{k}},\ldots ,1\right]}^{T}e^{-j2\varphi_{k}} \end{array} \end{array}$$

Combining $U_{1}^{\prime }$, $U_{2}^{\prime }$, and $U_{3}^{\prime }$ together, the output of the SDCA is formed as

(49) $$\begin{array}{c} \begin{array}{c} \begin{array}{cc} U & =\left[\begin{array}{c} U_{3}^{\prime }\\ U_{1}^{\prime }\\ U_{2}^{\prime } \end{array}\right]=\left[\begin{array}{c} C_{31}^{\prime }\\ C_{11}^{\prime }\\ C_{21}^{\prime } \end{array}\right]\left[p,\mathrm{Hp},\ldots ,H^{R_{1}}p\right]+\left[\begin{array}{c} 0\\ \sigma_{n}^{2}I\\ 0 \end{array}\right]\\ \; & =C_{e}\left[p,\mathrm{Hp},\ldots ,H^{R_{1}}p\right]+\left[\begin{array}{c} 0\\ \sigma_{n}^{2}I\\ 0 \end{array}\right] \end{array} \end{array} \end{array}$$

and its steering vector

(50) $$\begin{array}{c} \begin{array}{c} c_{e}\left(\theta_{k},\varphi_{k}\right)=\left[\begin{array}{c} c_{31}^{\prime }\left(\theta_{k},\varphi_{k}\right)\\ c_{11}^{\prime }\left(\theta_{k}\right)\\ c_{21}^{\prime }\left(\theta_{k},\varphi_{k}\right) \end{array}\right] \end{array} \end{array}$$

### 3.2. MSD-RD-MUSIC Algorithm

In order to estimate DOA based on the signal model (49), we calculate the covariance matrix as

(51) $$\begin{array}{c} \begin{array}{c} {\hat{R}}_{U}=\frac{1}{R_{1}+1}{\mathrm{UU}}^{H} \end{array} \end{array}$$

Eigenvalue decomposition [32] is performed on ${\hat{R}}_{U}$:

(52) $$\begin{array}{c} \begin{array}{c} {\hat{R}}_{U}=E_{s}\Lambda_{s}E_{s}^{H}+E_{n}\Lambda_{n}E_{n}^{H} \end{array} \end{array}$$

where $E_{s}$ and $E_{n}$ are signal and noise subspace, and the eigenvalues are collected in $\Lambda_{s}$ and $\Lambda_{n}$.

According to [3], a 2D-MUSIC cost function can be formed as

(53) $$\begin{array}{c} \begin{array}{c} F_{2D-\mathrm{MUSIC}}\left(\theta ,\varphi \right)=\frac{1}{c_{e}^{H}\left(\theta ,\varphi \right)E_{n}E_{n}^{H}c_{e}\left(\theta ,\varphi \right)} \end{array} \end{array}$$

To derive the RD-MUSIC cost function based on SDCA [24,26] and lower the computational complexity, we divide $c_{e}\left(\theta ,\varphi \right)$ into

(54) $$\begin{array}{c} \begin{array}{c} c_{e}\left(\theta ,\varphi \right)=A\left(\theta \right)g\left(\varphi \right)=\left[\begin{array}{ccc} a_{1}\left(\theta \right) & \;\; & \;\;\\ \;\; & a_{2}\left(\theta \right) & \;\;\\ \;\; & \;\; & a_{3}\left(\theta \right) \end{array}\right]\left[\begin{array}{c} e^{j2\varphi }\\ 1\\ e^{-j2\varphi } \end{array}\right] \end{array} \end{array}$$

where $a_{1}\left(\theta \right)={\left[e^{j\left(-R_{3}+R_{1}\right)\Delta \sin\theta },e^{j\left(-R_{3}+R_{1}+1\right)\Delta \sin\theta },\ldots ,1\right]}^{T}$, $a_{2}\left(\theta \right)={\left[1,e^{j\Delta \sin\theta },\ldots ,e^{jR_{1}\Delta \sin\theta }\right]}^{T}$, and $a_{3}\left(\theta \right)={\left[e^{jR_{1}\Delta \sin\theta },e^{j\left(R_{1}+1\right)\Delta \sin\theta },\ldots ,e^{jR_{3}\Delta \sin\theta }\right]}^{T}$. Substitute (54) into (53), the 2D-MUSIC function is rewritten as

(55) $$\begin{array}{c} \begin{array}{c} F_{2D-\mathrm{MUSIC}}\left(\theta ,\varphi \right)=\frac{1}{g^{H}\left(\varphi \right)A{\left(\theta \right)}^{H}E_{n}E_{n}^{H}A\left(\theta \right)g\left(\varphi \right)}=\frac{1}{g^{H}\left(\varphi \right)Q\left(\theta \right)g\left(\varphi \right)} \end{array} \end{array}$$

where $Q\left(\theta \right)=A{\left(\theta \right)}^{H}E_{n}E_{n}^{H}A\left(\theta \right)$. The trivial solution of the quadratic optimization problem is eliminated by constraint of $e_{x}^{H}g\left(\varphi \right)=1$, where $e_{x}={\left[0,1,0\right]}^{T}$. Thus, the optimization problem is reconstructed as

(56) $$\begin{array}{c} \begin{array}{c} \min_{\theta ,\varphi }g^{H}\left(\varphi \right)Q\left(\theta \right)g\left(\varphi \right),\;s.t.\;e_{x}^{H}g\left(\varphi \right)=1 \end{array} \end{array}$$

In handling the problem presented in (56), we define the cost function as follows:

(57) $$\begin{array}{c} \begin{array}{c} L\left(\theta ,\varphi \right)=g^{H}\left(\varphi \right)Q\left(\theta \right)g\left(\varphi \right)-\beta \left(e_{x}^{H}g\left(\varphi \right)-1\right) \end{array} \end{array}$$

where β is a constant. Its partial derivative satisfies

(58) $$\begin{array}{c} \begin{array}{c} \frac{\partial L\left(\theta ,\varphi \right)}{\partial g\left(\varphi \right)}=0 \end{array} \end{array}$$

Then,

(59) $$\begin{array}{c} \begin{array}{c} g\left(\varphi \right)=\mu Q^{-1}\left(\theta \right)e_{x}=\frac{Q^{-1}\left(\theta \right)e_{x}}{e_{x}^{H}Q^{-1}\left(\theta \right)e_{x}} \end{array} \end{array}$$

where μ is a constant. Substitute (59) into (55), the DOA of the source can be estimated as

(60) $$\begin{array}{c} \begin{array}{c} \hat{\theta }=\arg\max_{\theta }e_{x}^{H}Q\left(\theta \right)e_{x} \end{array} \end{array}$$

The main steps of the MSD-RD-MUSIC are summarized as follows:

Collect signals in the extended form in (12) and calculate its covariance matrix $R_{xe}$;
Separate $R_{xe}$ into four parts and vectorize each of them separately as described in (16)–(19);
Extract distinct rows from $u_{1}$, $u_{2}$, $u_{3}$ and form virtual signals of the SDCA;
Eliminate influence caused by synthetic parameter $\rho_{0}$ through (34)–(36), and construct the virtual signal matrix by spatial smoothing technique in (49);
(5) Calculate the sampling covariance matrix ${\hat{R}}_{U}$, and perform eigenvalue decomposition by (52) to obtain the noise subspace $E_{n}$;
(6) Finally, estimate the DOAs of the sources via RD-MUSIC in (60).

## 4. Performance Analysis of Proposed Algorithm

We analyze the DOFs and computational complexity of our algorithm and compare it with other competitors to highlight its superiority. The method based on RD-MUSIC proposed in [24] is customized for CD sources with the vectorization procedure introduced in Section 3 and is referred to as SD-RD-MUSIC. TLS-ESPRIT-CD [11] based on ULA and generalized ESPRIT [13] based on symmetrical ULA modified for noncircular signals are denoted by NC-ESPRIT-CD and NC-SSI-CD for short, respectively. The 2D-MUSIC algorithm introduced in (53) and the method designed for circular CD sources in [27] are referred to as MSD-2D-MUSIC and NSS-MUSIC.

### 4.1. Degrees of Freedom

We analyze the DOFs of all competitors in this part.

Firstly, for MSD-RD-MUSIC and MSD-2D-MUSIC, the DCA and two SCAs are leveraged, so the index position of the virtual ULA ranges from $\left(-R_{3}+R_{1}\right)$ to $R_{3}$. As a result, we have ${\mathrm{DOF}}_{\mathrm{MSD}-\mathrm{RD}-\mathrm{MUSIC}}={\mathrm{DOF}}_{\mathrm{MSD}-2D-\mathrm{MUSIC}}=2R_{3}-R_{1}+1$.

For SD-RD-MUSIC [24], only one DCA and one SCA are employed. Thus, its DOFs are slightly smaller than our method, and we know that ${\mathrm{DOF}}_{\mathrm{SD}-\mathrm{RD}-\mathrm{MUSIC}}=R_{3}+1$.

Thirdly, for NSS-MUSIC [27], initially designed for circular signals, only the DCA could be used. So, ${\mathrm{DOF}}_{\mathrm{NSS}-\mathrm{MUSIC}}=R_{1}+1$.

Finally, NC-ESPRIT-CD and NC-SSI-CD are both based on ULAs. Note that only sensors in one subarray are counted for NC-ESPRIT-CD. Thus, their DOFs are twice the number of the sensors, which means ${\mathrm{DOF}}_{\mathrm{NC}-\mathrm{ESPRIT}-\mathrm{CD}}={\mathrm{DOF}}_{\mathrm{NC}-\mathrm{SSI}-\mathrm{CD}}=2N$.

We assume that the number of sensors utilized in these algorithms is the same. For $N_{1}⩾3$ and $N_{2}>3$, the following conclusion is drawn: ${\mathrm{DOF}}_{\mathrm{MSD}-\mathrm{RD}-\mathrm{MUSIC}}={\mathrm{DOF}}_{\mathrm{MSD}-2D-\mathrm{MUSIC}}>$ $\;{\mathrm{DOF}}_{\mathrm{SD}-\mathrm{RD}-\mathrm{MUSIC}}>{\mathrm{DOF}}_{\mathrm{NSS}-\mathrm{MUSIC}}>{\mathrm{DOF}}_{\mathrm{NC}-\mathrm{ESPRIT}-\mathrm{CD}}={\mathrm{DOF}}_{\mathrm{NC}-\mathrm{SSI}-\mathrm{CD}}$.

### 4.2. Computational Complexity

Computational complexity in this paper mainly refers to the complex multiplication times of the algorithms, and the following parameters are involved: number of sensors $N=N_{1}+N_{2}$ (for NC-SSI-CD [13], the sensor positions of symmetrical ULA are from −M to *M*, and there is N=2M+1), number of snapshots *L*, number of sources *K*, the searching range of DOA is separated into $\alpha_{\theta }$ equal parts, and the range of the noncircular phase is divided into $\alpha_{\varphi }$ equal parts.

For our method, computation of the covariance matrix ${\hat{R}}_{x_{e}}$ costs $O\left(LN^{2}\right)$. Multiplication of the reverse of diagonal matrices in (34)–(36) takes $O\left(2R_{1}+2R_{3}+3\right)$. Calculation of the covariance matrix ${\hat{R}}_{U}$ requires $O(\left(R_{1}+1\right){\left(2R_{3}-R_{1}+3\right)}^{2})$. Eigenvalue decomposition for ${\hat{R}}_{U}$ needs $O({\left(2R_{3}-R_{1}+3\right)}^{3})$. For the peak-searching procedure, $O\left(\alpha_{\theta }\left(3\left(2R_{3}-R_{1}+3\right)\left(2R_{3}-R_{1}+3-K\right)+9\left(2R_{3}-R_{1}+3-K\right)\right)\right)$ is needed to estimate the DOAs of the sources.

**Figure 3:**
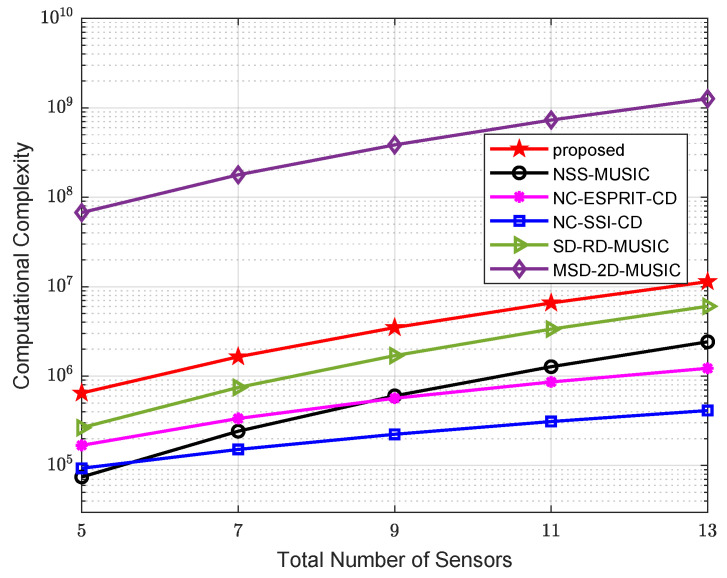
Comparison of complexity versus number of sensors.

**Figure 4:**
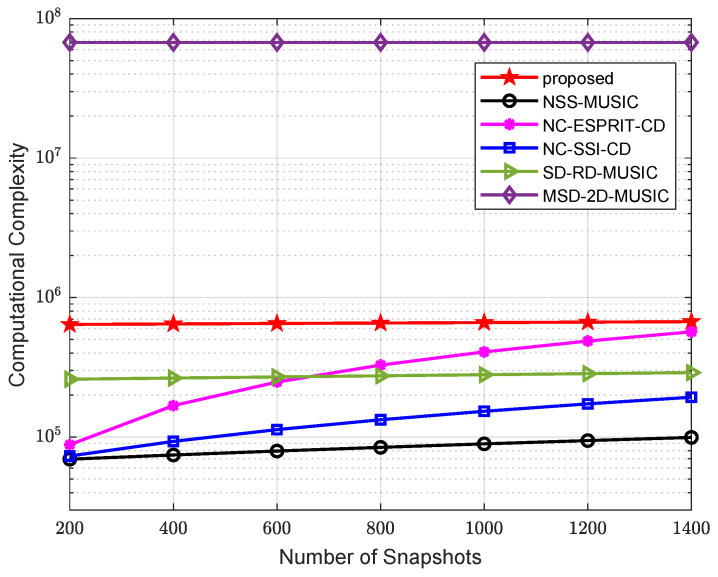
Comparison of complexity versus number of snapshots.

The complex multiplication times for the rest algorithms are also calculated. For clarity, the complexity of all algorithms is summarized in Table 1. A comparison of complex multiplication times versus the total number of sensors is presented in Figure 3, where K=2 sources are scattered in space, the snapshot number is 400, and there are $\alpha_{\theta }=180/0.2=900$ and $\alpha_{\varphi }=180/0.5=360$ searching grid points for DOA and the noncircular phase, and each search range is from −90° to 90°. A comparison of complexity versus the number of snapshots is illustrated in Figure 4, where there are a total of 5 sensors ($N_{1}=2$, $N_{2}=3$, M=2) in the array, and the rest of the parameters are the same. Compared with MSD-2D-MUSIC, our algorithm only performs a one-dimensional peak-searching program, thus reducing computational complexity significantly. Compared with the SD-RD-MUSIC and NSS-MUSIC, its complexity is slightly higher because a longer virtual array is constructed in this algorithm. Compared with ULA-based low-complexity methods, i.e., NC-ESPRIT-CD and NC-SSI-CD, a higher number of DOFs and accuracy is achieved by our proposed algorithm, and therefore, it has higher complexity.

### 4.3. Advantages of the Proposed Algorithm

The advantages of MSD-RD-MUSIC are summarized as follows:

1. Comparing MSD-2D-MUSIC with the cost function introduced in (53), our algorithm maintains the same DOFs while reducing complexity remarkably, which strikes a balance between the performance and complexity.
2. Compared with SD-RD-MUSIC [24], it makes use of all three co-arrays. The extra positive SCA can provide additional DOFs, which improves the estimation accuracy.
3. For algorithms based on ULAs [11,13], their maximum DOFs are only twice the length of the array, which is much smaller than the DOFs of MSD-RD-MUSIC.

## 5. Numerical Simulation Results

In this section, several examples are given to illustrate the performance and advantages of our algorithm concretely. The calculation of root mean squared error (RMSE) is defined as

(61) $$\begin{array}{c} \begin{array}{c} \mathrm{RMSE}=\frac{1}{K}\sum_{k=1}^{K}\sqrt{\frac{1}{M_{t}}\sum_{m_{t}=1}^{M_{t}}{\left({\hat{\theta }}_{k,m_{t}}-\theta_{k}\right)}^{2}} \end{array} \end{array}$$

where $M_{t}$ is the number of Monte Carlo trials, ${\hat{\theta }}_{k,m_{t}}$ is the estimated direction of the *k*th emitter in the $m_{t}$th trial, and $\theta_{k}$ is the true direction of the *k*th emitter. Except for the first example, the total number of the Monte Carlo trials is 500. We also assume that $\rho_{0}=0.995$ for all sources in simulations. Unit intersensor spacing $d_{0}$ is set to λ/4 in order to eliminate phase ambiguity in NC-SSI-CD.

**Figure 5:**
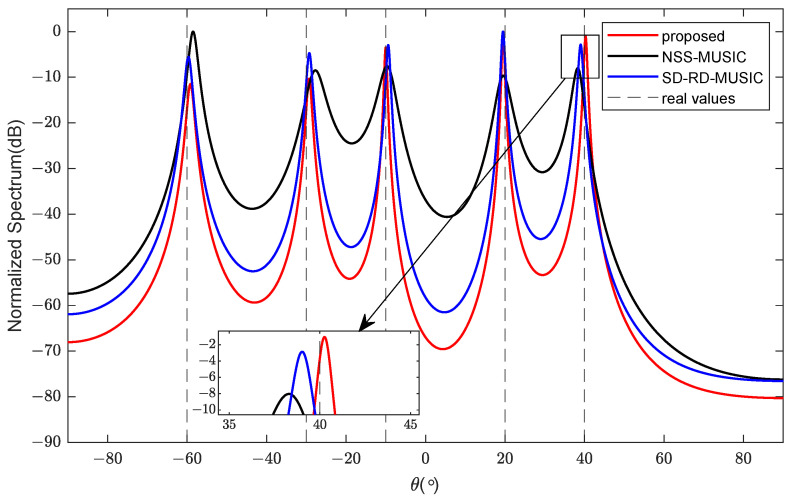
Comparison of spectrums.

In the first example, we plot the spectrums of MSD-RD-MUSIC, SD-RD-MUSIC, and NSS-MUSIC in Figure 5. The number of sources is K=5, and DOAs are −60°, −30°, −10°, 20°, and 40°. There are a total of five sensors (M=2, $N_{1}=2$, $N_{2}=3$) in the NA. Angular spreads and noncircular phases are set to be 4° and 10°. L=1000 snapshots are provided for the direction estimation. The signal-to-noise ratio (SNR) is set to 10 dB. It is shown in the figure that the spectrums of MSD-RD-MUSIC and SD-RD-MUSIC are very close to each other and both behave better than that of NSS-MUSIC.

**Figure 6:**
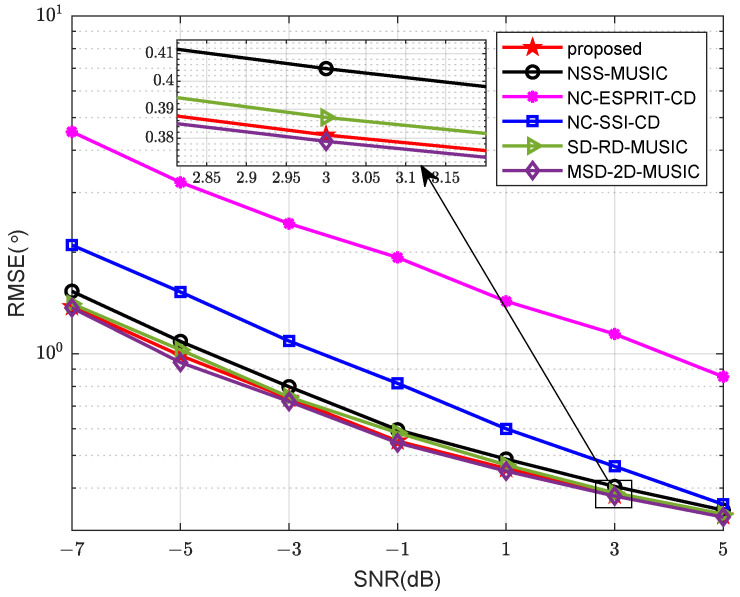
Comparison of RMSE versus SNR.

In the second example, we compare the RMSE of different algorithms versus SNR. We assume that there are two sources in the space, which are located in the direction of −40° and 40°. The noncircular phases are 10° and 15°, and spreads of sources are both 4°. The number of snapshots is set to L=600. The number of sensors is N=5 (M=2, $N_{1}=2$, $N_{2}=3$). The result is shown in Figure 6. We can learn that the accuracy of the proposed algorithm and MSD-2D-MUSIC are very close, which means that the reduced-dimension strategy based on the idea of RD-MUSIC can lower computational complexity with little performance deterioration. Our method behaves slightly better than SD-RD-MUSIC and NSS-MUSIC because more DOFs are obtained. All the algorithms based on sparse arrays have better performance than those based on ULAs.

**Figure 7:**
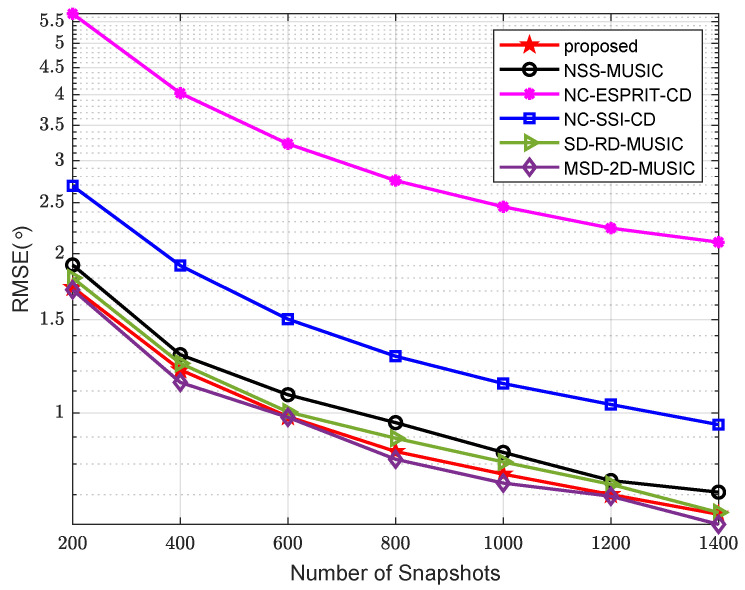
Comparison of RMSE versus number of snapshots.

In the third example, a comparison of the RMSE of algorithms versus the number of snapshots is made, where SNR = −5 dB, and other parameters are the same as those in the second example. The result is shown in Figure 7. There is no doubt that MSD-2D-MUSIC has the best performance due to its high complexity. Excluding that, our algorithm yields the best result.

**Figure 8:**
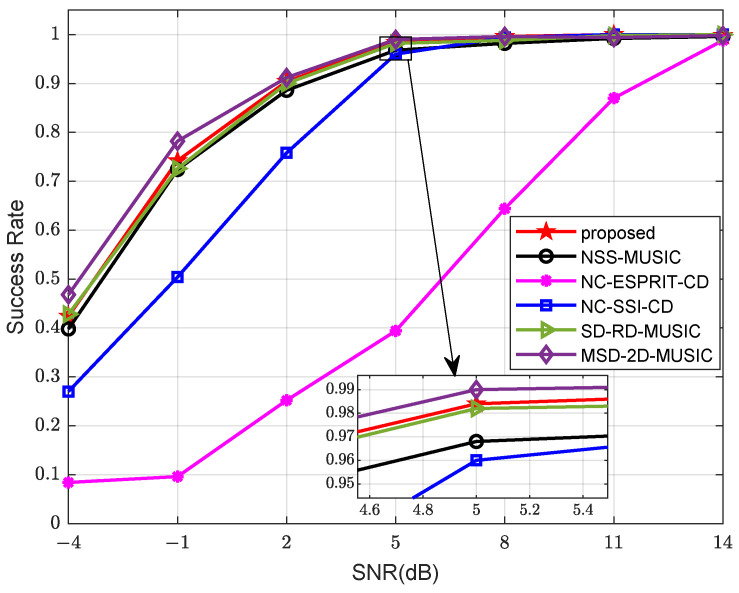
Comparison of success rate versus SNR.

**Figure 9:**
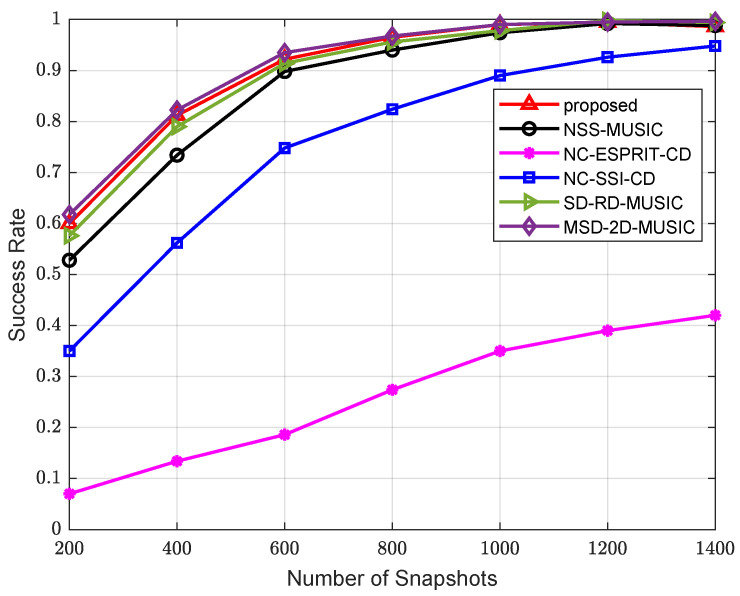
Comparison of success rate versus number of snapshots.

Finally, we run tests on the success rate of the algorithms. The results are presented in Figure 8 and Figure 9. Source and array parameters remain unchanged from those in the second and third examples. An estimation is considered successful when the deviations of the results are both less than 1°. The calculation of the success rate $P_{sr}$ is given as

(62) $$\begin{array}{c} \begin{array}{c} P_{sr}=\frac{T_{v}}{T_{t}} \end{array} \end{array}$$

where $T_{v}$ is the number of valid estimation results, and $T_{t}$ is the total times of trials. In Figure 8, 400 snapshots are involved. It is clearly shown that MSD-2D-MUSIC with a two-dimensional peak-searching procedure has the highest success rate. The proposed algorithm has the second best performance, with its success rate reaching 100% at approximately 8 dB. In Figure 9, the SNR is fixed at 0 dB. The conclusion is essentially the same as the simulation in Figure 8. The success rate of algorithms based on NAs reaches 100% at about 1200 snapshots.

## 6. Conclusions

In this paper, a nested array processing method for noncircular CD sources is presented. Similarity among sources is well utilized to approximate these CD sources into conventional point sources in order to perform the SS technique to restore the rank. The proposed algorithm makes use of all the virtual sensors generated. Thus, compared with other algorithms, it has the most DOFs. Then, we lower the computational complexity of the algorithm by designing a variant of the RD-MUSIC cost function. Numerical simulations prove that the proposed algorithm has perfect direction-finding accuracy with the same parameters. In the future, more research could be conducted to develop advanced signal parameter estimation methods. Different array configurations could also be considered. It might be possible for future methods to remove some limitations in the current study.

## Figures and Tables

**Table 1:** Complexity of algorithms.

Algorithm	Computational Complexity
proposed MSD-RD-MUSIC	$O(LN^{2}+2R_{1}+2R_{3}+3+\left(R_{1}+1\right){\left(2R_{3}-R_{1}+3\right)}^{2}+{\left(2R_{3}-R_{1}+3\right)}^{3}+\alpha_{\theta }\left(3\left(2R_{3}-R_{1}+3\right)\left(2R_{3}-R_{1}+3-K\right)+9\left(2R_{3}-R_{1}+3-K\right)\right))$
MSD-2D-MUSIC	$O(LN^{2}+2R_{1}+2R_{3}+3+\left(R_{1}+1\right){\left(2R_{3}-R_{1}+3\right)}^{2}+{\left(2R_{3}-R_{1}+3\right)}^{3}+\alpha_{\theta }\alpha_{\varphi }\left(\left(2R_{3}-R_{1}+3\right)\left(2R_{3}-R_{1}+3-K\right)+\left(2R_{3}-R_{1}+3-K\right)\right))$
NSS-MUSIC [27]	$O(LN^{2}+\left(2R_{1}+1\right)+2{\left(R_{1}+1\right)}^{3}+\alpha_{\theta }\left(R_{1}+1\right)\left(R_{1}+1-K\right)+R_{1}+1-K)$
SD-RD-MUSIC [24]	$O(LN^{2}+2R_{1}+R_{3}+2+\left(R_{1}+1\right){\left(R_{3}+2\right)}^{2}+{\left(R_{3}+2\right)}^{3}+\alpha_{\theta }\left(2\left(R_{3}+2\right)\left(R_{3}+2-K\right)+4\left(R_{3}+2-K\right)\right))$
NC-ESPRIT-CD [11]	$O(L{\left(4N\right)}^{2}+{\left(4N\right)}^{3}+8NK^{2}+11K^{3})$
NC-SSI-CD [13]	$O(L{\left(2N\right)}^{2}+{\left(2N\right)}^{3}+\alpha_{\theta }\left(2N+K^{3}+2NK^{2}\right))$

## Data Availability

The data used in this paper can be requested from the corresponding authors upon request.
